# Regulation of Oil Biosynthesis and Genetic Improvement in Plants: Advances and Prospects

**DOI:** 10.3390/genes15091125

**Published:** 2024-08-26

**Authors:** Lixia Zhou, Qiufei Wu, Yaodong Yang, Qihong Li, Rui Li, Jianqiu Ye

**Affiliations:** 1 National Key Laboratory for Tropical Crop Breeding, Chinese Academy of Tropical Agricultural Sciences, Haikou 571101, Chinayyang@catas.cn (Y.Y.); liqihong@catas.cn (Q.L.); lirui@catas.cn (R.L.); 2 Coconut Research Institute, Chinese Academy of Tropical Agricultural Sciences, Wenchang 571339, China

**Keywords:** seed oil, synthesis metabolism, key enzyme, transcription factor, co-regulation

## Abstract

Triglycerides are the main storage form of oil in plant seeds. Both fatty acids and triglycerides possess important functions in the process of plant growth and development. To improve the seed oil content and improve its fatty acid composition, this paper analyzed the research progress on the oil regulation and synthesis metabolism process of plant seeds and summarized the strategies for the improvement of plant seed oil: (a) To regulate carbon distribution by inhibiting the expression of genes encoding key enzymes, allocating carbon sources into the protein synthesis pathway, and enhancing the expression of key genes encoding key enzymes, leading carbon sources into the synthesis pathway of fatty acids; (b) To intervene in lipid synthesis by promoting the biosynthesis of fatty acids and improving the expression level of key genes encoding enzymes in the triacylglycerol (TAG) assembly process; (c) To improve seed oil quality by altering the plant fatty acid composition and regulating the gene expression of fatty acid desaturase, as well as introducing an exogenous synthesis pathway of long chain polyunsaturated fatty acids; (d) To regulate the expression of transcription factors for lipid synthesis metabolism to increase the seed oil content. In addition, this article reviews the key enzymes involved in the biosynthesis of plant fatty acids, the synthesis of triacylglycerol, and the regulation process. It also summarizes the regulatory roles of transcription factors such as *WRI*, *LEC*, and *Dof* on the key enzymes during the synthesis process. This review holds significant implications for research on the genetic engineering applications in plant seed lipid metabolism.

## 1. Introduction

Fatty acid metabolism in plant bodies is one of the fundamental metabolic processes that sustain their life activities and also provide humans with an important source of energy [1,2]. Since the human body cannot synthesize certain essential fatty acids on its own, consuming plant oils extracted from seeds has become one of the primary ways to obtain these essential fatty acids [3]. The fatty acids stored in plant seeds are often present in the form of triacylglycerols (TAGs)—that is, glycerol molecules with three fatty acids attached [4]. Oils are the primary form of energy storage in plant seeds, providing indispensable energy for seed germination and the initial growth of seedlings. Beyond their use in food, fatty acids also have significant value in industrial production, serving as raw materials for the manufacture of paints, surfactants, lubricants, nylon, pharmaceuticals, and more [5]. Therefore, research aimed at improving the content and composition of fatty acids in plant seeds to meet both dietary and industrial needs is increasingly garnering attention. One of the tasks in crop breeding is to develop new varieties of oil-seed crops with a higher oil content and a healthier ratio of unsaturated fatty acids.

**Figure 1:**
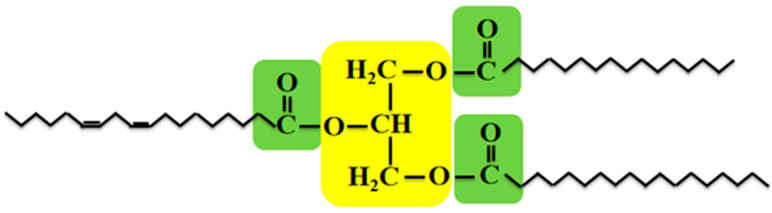
Chemical structure of TAG. The ester linkages are highlighted in green, and the glycerol backbone is in yellow. The bending lines represent fatty acid chains, and two short lines in the sn-2 position indicate unsaturated bonds.

The metabolic pathways of plant lipid synthesis are indeed complex. During seed development, sucrose serves as the primary carbon source for fatty acid synthesis, transported from photosynthetic organs (such as leaves) to the seed cells [6]. Through plant glycolysis, significant amounts of precursors for TAG synthesis are produced, including dihydroxyacetone phosphate and pyruvate. Pyruvate undergoes oxidative decarboxylation to form acetyl-CoA, which is then transported to the plastids for de novo fatty acid synthesis [7]. The synthesized fatty acids are subsequently transported to the endoplasmic reticulum where they are assembled with glycerol 3-phosphate to form TAGs. These synthesized TAGs are finally transported to oil bodies for storage. TAGs are a type of glyceride, with a backbone composed of glycerol, where the three hydroxyl groups on the glycerol are each connected to fatty acids through esterification (Figure 1) [8]. The carbon atoms in the fatty acids are in a highly reduced state, making them an efficient form of carbon source and energy storage. From an evolutionary perspective in plants, lipids are beneficial for meeting the material and energy needs of seed germination and subsequent seedling growth; from an economic standpoint, the amount of lipids in the seeds of economic crops is a determining factor in yield [9]. Therefore, the pathways and regulatory mechanisms of lipid synthesis in plants have garnered widespread attention. This intricate physiological and biochemical process is regulated by various functional enzymes and transcription factors, which have been reported in crops such as *Arabidopsis*, rapeseed, soybean, maize, and cotton [10,11,12,13]. Key enzyme genes like *ACCase*, *DGAT*, and *FAS*, as well as transcription factors such as *LEC*, *WRI*, and *Dof*, are among the hotspots of research [14,15,16]. This review summarized the progress in the study of key enzymes and transcription factors involved in the regulation of lipid synthesis metabolism in plant seeds, providing insights and references for the improvement of plant oil quality, including their importance for human health [17].

The research progress on some key enzymes and transcription factors involved in the process of plant oil synthesis was reviewed. Strategies for improving plant oil content and quality through genetic engineering techniques were discussed and an outlook on the genetic improvement pathways for plant oils was provided.

## 2. Identification and Selection of Literature

We will use a forward citation tracing approach to identify the relevant literature. We will conduct our search in the Web of Science (WOS) database, which was developed for citation analysis and indexes journals broadly across the health and social science disciplines that publish implementation research. Because there could be delays in archiving more recent works in WOS, we will also draw on citation alerts sent to the second author (EKP) from the publisher for a 6-month period coinciding with the WOS citation search. Citations will be managed using Mendeley and exported to Covidence, a web-based program designed to manage references for systematic and scoping reviews, for deduplication.

Articles will be screened for inclusion in a two-phase process. First, two independent screeners will review each article title and abstract and apply the inclusion and exclusion criteria. Articles will be included if they (a) report the results of an empirical study and (b) are published in a peer-reviewed journal. These inclusion criteria are intended to include articles reporting on instrument or measurement development studies and using a diverse range of methodologies (e.g., quantitative, qualitative, or mixed).

Articles will be excluded if they (a) do not report the results of an empirical study (e.g., editorials, commentaries, study protocols, summaries, narrative reviews, “lessons learned”), (b) are not published in a peer-reviewed journal (e.g., books, book chapters, reports, monographs, magazines, websites/blogs, newsletters), or (c) report on the results of a systematic review. However, if we locate a systematic review focused on the measurement or evidence of implementation outcomes, we will locate and consider the studies included in those reviews. Discrepancies in screening decisions will be reviewed by two team members who will reach consensus on a decision.

## 3. Process of Oil Synthesis and Function of Related Genes

**Figure 2:**
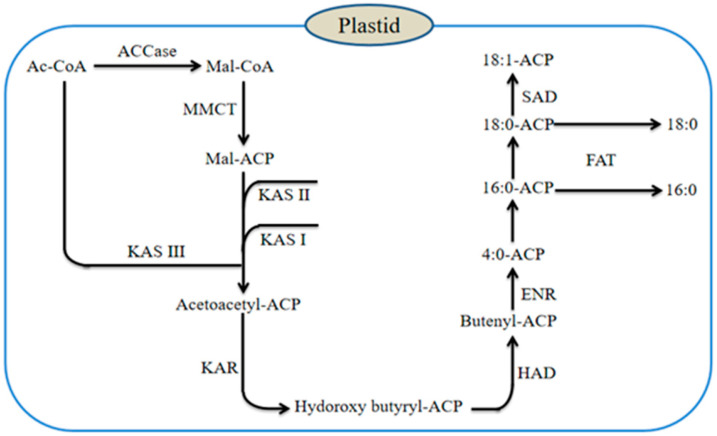
Diagram of fatty acid biosynthesis.

Fatty acid is the main component of oil, and its synthesis is mainly carried out in plastids [18]. Firstly, acetyl CoA carboxylase (ACCase) catalyzes acetyl CoA to form malonyl CoA (Mal-CoA), and then the fatty acid synthase (FAS) system extends the carbon chain through condensation, reduction, dehydration, and re-reduction (Figure 2) [19]. The FAS system is a multi-enzyme complex, including acyl carrier protein (ACP) and many kinds of enzymes. All catalytic reactions are carried out on ACP. FAS catalyzes continuous cycle polymerization, adding two carbon acyl carbon chains in each cycle until the synthesis of saturated fatty acids palmitic acid (16:0-ACP) and stearic acid (18:0-ACP). Unsaturated fatty acids are formed under the action of desaturase, including monounsaturated fatty acids such as palmitoleic acid and oleic acid, and long-chain polyunsaturated fatty acids such as linoleic acid and linolenic acid. The fatty acids are then released from ACP by acyl-ACP thioesterase (FAT). FAT can be divided into FATA and FATB according to the different substrates, they have carbon chain length specificity, and their activity affects the composition of fatty acid [20,21].

Enzymes involved in the biosynthesis of plant seed oil include key enzymes and transcription factors that affect carbon source allocation and participate in FA synthesis and TAG assembly, etc. [22]. The function of related genes is summarized in Table 1.

## 4. Key Enzymes Affecting Carbon Source Distribution

### 4.1. Phosphoenolpyruvate Carboxylase, PEPCase

Phosphoenolpyruvate carboxylase (PEPCase) is a cytoplasmic enzyme that catalyzes the formation of oxaloacetate (OAA) from phosphoenolpyruvate (PEP) and HCO_3_^−^ [46]. OAA is involved in the tricarboxylic acid cycle (TCA), affecting the distribution of substrate and increasing oil content [47]. There is a significant negative correlation between protein content and oil content in plant seeds. A high activity of PEPCase can promote more carbon sources towards the protein biosynthesis pathway and inhibit carbon sources from the oil synthesis pathway. The oil content in the Brassica seeds of the transformed PEPCase antisense expression vector was more than 15% higher than that of the control [48]. Transgenic plants were also obtained from rice, soybean, and peanut by using substrate competition to regulate the ratio of protein and oil in grains and to construct the antisense plant expression vector of the PEPCase gene. PEPCase 1 and 2 have been cloned from cotton and are involved in the regulation of cotton fiber elongation [23]. The cottonseed oil content in several transgenic lines showed a significant increase (up to 16.7%) without obvious phenotypic changes under field conditions when compared to the control plants by the downregulation of phosphoenolpyruvate carboxylase 1 (*GhPEPC1*) via RNA interference in transgenic cotton plants. The expression of the carbon metabolism-related genes of transgenic GhPEPC1 RNAi lines was quantified by transcriptome analysis. The analysis revealed that the decrease in *GhPEPC1* expression led to increased expression of triacylglycerol biosynthesis-related genes, which eventually contributed to lipid biosynthesis in cotton [24].

### 4.2. Pyruvate Dehydrogenase Complex, PDHC

The pyruvate dehydrogenase complex (PDHC) is a multi-enzyme complex composed of the pyruvate dehydrogenase component (E1), dihydrolipoyl transacetylase (E2), and dihydrolipoyl dehydrogenase (E3). E1 comprises two subunits, E1-α and E1-β. PDHC competes with PEPCase for pyruvic acid and catalyzes its oxidative decarboxylation to form acetyl-CoA [49].

PDHC catalyzes the synthesis of acetyl-CoA from pyruvic acid, which is not only the entrance material for the TAC cycle but also a precursor for FA synthesis. Pyruvic acid decomposition provides energy for lipid synthesis and metabolism, and its decomposition product serves as a precursor for lipid biosynthesis. PDHC is present in mitochondria and chloroplasts, but their activities differ. The content of acetyl-CoA in the chloroplasts of pea and spinach leaves was low, and they could hardly maintain fatty acid synthesis. However, plastid PDHC could rapidly produce acetyl-CoA in large quantities, providing a substrate for fatty acid synthesis. The activity of PDHC in the mitochondria of pea was three times higher than that in the chloroplast [25]. The expression level of PDHC was significantly higher than that of the acetyl-CoA synthase gene during oil accumulation in Arabidopsis seeds. Inhibition of the E1-α subunit activity of PDHC in tapetum cells of sugar beet resulted in abnormal development of the microspore exine and male sterility [26].

## 5. Key Enzymes in De Novo Fatty Acid Synthesis

### 5.1. Acetyl-CoA Carboxylase, ACCase

Acetyl-CoA carboxylase (ACCase) is one of the key enzymes in fatty acid biosynthesis. ACCase catalyzes the carboxylation of acetyl-CoA to form malonyl-CoA in plant seeds, which is a key regulatory step in fatty acid synthesis and oil formation, and ACCase is also an important gene affecting the entire life process of plants [50,51].

ACCase can be divided into two types: heterotype and homogeneous type. In the dicotyledons and monocotyledons of non-gramineous plants, ACCase is heterogeneous and consists of four subunits: biotin carboxyl carrier protein (BCCP), biotin carboxylase (BC), and the carboxytransferase α and β subunits (α-CT and β-CT) [52]. Homologous ACCase exists in the cytoplasm of plants. Each subunit of ACCase has all the catalytic functions of ACCase, but it can only be activated when they are polymerized into a complete enzyme [53]. It also exists in the cytoplasm of algae, yeast, animals, and some plants [54]. In most higher plants, malonyl-CoA catalyzed by homomorphic ACCase is used for fatty acid chain extension and the synthesis of many secondary metabolites, such as flavonoids, while the heterotype is used for the de novo biosynthesis of fatty acids [55].

Research indicates that the rates of fatty acid synthesis and oil accumulation are closely related to the activity of ACCase. For example, the activity of ACCase in high-oil soybean is twice higher than in low-oil soybean from the early to middle stage of seed development [27], and the expression level of low-oil content mutant WRINKLED1 ACCase in Arabidopsis is significantly lower than that in wild type [56]. In oilseed rape, the seed-specific promoter was fused with the *Arabidopsis* homologous ACCase gene and then transferred by soybean transfer peptide. When ACCase was introduced into rape chloroplast, the ACCase activity of mature seeds of transgenic rape was 10–20 times higher than that of the control, and the oil content increased by 5% [30]. Because the structure of the heterotype is more complex than that of homomorphism, the research on heterotype is relatively less. It was found that ACCase reaction activity, BC, and BCCP expression levels were correlated with oil accumulation in Castor seeds [57]. Overexpression of the carboxyl-transferase B subunit (accD) in plastids of various tissues resulted in an increase in fatty acid content in the leaves of transgenic plants and the obvious growth of plant leaves. Although the fatty acid content of seeds of transgenic progenies did not change significantly compared to that of the wild type, the seed yield of transgenic progeny increased nearly two times, thus increasing the oil content of single plant seeds.

### 5.2. Key Enzymes for Carbon Chain Extension

#### 5.2.1. Ketoacyl-ACP Synthase, KAS

Ketoacyl-ACP synthase (KAS) consists of three types of enzymes: KASI, KASII, and KASIII. KASIII catalyzes the formation of 4:0-ACP, KASI catalyzes the extension of the carbon chain from 4:0-ACP to 16:0-ACP, and KASII catalyzes the formation of 16:0-ACP to 18:0-ACP [58,59,60].

The fatty acid composition can be changed by controlling the expression of the KASIII gene. For example, after the deletion of the KASIII and FATB (acyl-ACP thioesterase B, FATB) genes in Brassica [31], the fatty acid composition can be changed. The content of medium-chain fatty acids in the mutant was increased. KASI plays an important role in the formation of fatty acids. The oil content and plant fertility of AtKASI mutant seeds were significantly reduced. The oil content and fatty acid composition of the mutant could be restored by complementary tests. KASII mutation could change the fatty acid composition of plant seeds: the C16 fatty acid content increased and the C18 fatty acid content decreased. For example, the complementary expression of the JcKASII gene in the KASII mutant of Arabidopsis can restore the wild-type phenotype [32].

#### 5.2.2. Hydroxyacyl-ACP Dehydrase (HAD), Enoyl-ACP Reductase (ENR), β-Ketoacyl-ACP Reductase (KAR)

Hydroxyacyl-ACP dehydrase (*HAD*), Enoyl-ACP reductase (*ENR*), and β-ketoacyl-ACP reductase (*KAR*) are genes related to de novo fatty acid synthesis and play an important role in carbon chain extension [61,62]. During seed development, the expression levels of *KAR* and *ENR* reached their highest value on the 25th day after anthesis, and then decreased, while *HAD* showed an irregular trend. It was speculated that *KAR* and *ENR* may be required for fatty acid synthesis, and *HAD* may play different functions. The full-length cDNA of the *GhKAR*, *GhHAD*, and *GhENR* genes in cotton has been cloned. Overexpression of the *GhKAR* and *GhENR* genes significantly increased the oil content of cottonseed, the content of unsaturated fatty acids in cottonseed, and the cold resistance of transgenic lines [35]. During pericarp ripening, the expression levels of the *KAR*, *HAD*, and *ENR* genes in oil palm were significantly higher than those in Date palm, and the expression levels of *KAR* in oil palm were 44 times higher than those in Date palm [33]. The expression levels of *KAR*, *HAD*, and *ENR* in the leaves of peanut were significantly higher than those in roots and stems [34].

### 5.3. Fatty Acid Desaturase

Saturated fatty acids are desaturated by acyl-ACP desaturase to form monounsaturated or polyunsaturated fatty acids. Stearoyl-ACP desaturase (SAD) and fatty acid desaturase (FAD) are the most studied acyl-ACP desaturases. SAD catalyzes the desaturation of stearic acid to produce oleic acid, and then the oleic acid is transferred to the thylakoid membrane or into the cytoplasm for further desaturation. To further desaturate to form polyunsaturated fatty acids, FAD is required. SAD and FAD are two key enzymes that determine the content of different fatty acids [49].

Oil quality can be improved by regulating the expression of SAD and FAD coding genes. Some studies have shown that *FAD2* silencing can significantly increase the content of oleic acid in plants. Specifically, the content of oleic acid in Brassica and *Brassica juncea* increased to 89% and 75%, respectively [63]. Researchers have successfully constructed a seed-specific promoter NAPIN-regulated ihpRNA expression vector and artificial miRNA expression vector targeting *GhFAD2-1* gene in cotton [64]. The stearic acid content can be increased by inhibiting SAD gene expression; it has been reported in cotton that the stearic acid content of cottonseed was increased from 2% to 40% and the oleic acid content was increased from 15% to 77% by using interference technology [65]. SAD can affect FA composition and enhance plant resistance, such as the overexpression of Δ9 dehydrogenase of two wild-type potatoes in the plastid of tobacco, through which the fatty acid composition was changed, the content of unsaturated fatty acid in the leaves and seeds was increased, and the cold tolerance of the plant was improved [66]. The full-length cDNA of the *GhSAD2* gene in cotton has been obtained by homologous cloning, but its effects on fatty acid accumulation and cold resistance need to be further studied [67].

### 5.4. Diacylglycerol Acyltransferase (DGAT)

Diacylglycerol acyltransferase (DGAT) is a rate-limiting enzyme in the synthesis of triacylglycerol. Diacylglycerol is combined with fatty acid acyl to form triacylglycerol [68]. In 1998, the cDNA of *DGAT* was found in mice for the first time, and then the cDNA of DGAT1 was cloned in Arabidopsis. Two homologous genes, *DGAT2A* and *DGAT2B*, of DGAT2 were also cloned in *Mortierella ramanniana* [69,70,71].

There are three types of DGAT: *DGAT1*, *DGAT2*, and *DGAT3*. *DGAT1* is a key enzyme in the oil synthesis of plant seeds. The overexpression of *AtDGAT1* makes the DGAT activity of transgenic Arabidopsis seeds 10% to 70% higher than that of the wild type, and the thousand seed weight and oil content of seeds are also higher than those of the wild type [36]. *DGAT2* is considered to be the main gene for accumulating a large number of special fatty acids (such as ricinoleic acid), but *DGAT2* is also involved in the accumulation of conventional TAG in olive and oil palm [33]. The expression of short chain *s-NcDGAT2* of Neurospora in maize increased the oil content, changed the composition of fatty acids, increased the content of oleic acid, and decreased the content of linoleic acid [40].

The research on DGAT in regulating lipid synthesis mainly focuses on the *DGAT1* and *DGAT2* gene family. Although the protein sequences of these two *DGATs* are different, they both have the function of catalyzing diacylglycerol to form triacylglycerol by acyl-CoA. Generally speaking, in most plants, *DGAT1* plays a more widespread role in the synthesis and metabolism of triacylglycerol, while *DGAT2* focuses on the accumulation of special fatty acids, and their roles are not mutually exclusive [72]. Wurie et al. found that *DGAT2* acted upstream of the *DGAT1* gene and affected TAG synthesis and storage [73]. The results showed that the expression of *DGAT* affected seed development, oil content, fatty acid composition, and seed weight [74].

### 5.5. Acyl-ACP Thioesterase, FAT

Acyl-ACP thioesterase (FAT) catalyzes the final step in fatty acid synthesis, determining the type and chain length of newly synthesized free fatty acids. FAT transfers the acyl group synthesized on ACP to CoA or directly releases it from ACP to form free fatty acids [75]. In plants, there are mainly two types of FAT: FATB and FATA, which have substrate specificity for carbon chain lengths.

FATB primarily participates in the release of saturated acyl chains and to a lesser extent in the formation of unsaturated acyl chains. The most common is the 16:0-ACP thioesterase, which catalyzes the production of C16:0. Additionally, in plants rich in medium-chain fatty acids such as coconut, palm, and oil palm, there exist C8:0-ACP thioesterase, C10:0-ACP thioesterase, C12:0-ACP thioesterase, and C14:0-ACP thioesterase [76]. FATA primarily encodes the C18:1-ACP thioesterase and can also encode the C18:0-ACP thioesterase, exhibiting strong C18:1-ACP activity and relatively weak C18:0-ACP activity. FAT exhibits varying enzyme activities towards different substrates: it has a low activity towards stearoyl-ACP and palmitoyl-ACP, a higher activity towards unsaturated palmitoyl-ACP, and the highest activity towards oleoyl-ACP [77]. The isolation of a FATB-type laurate ACP thioesterase gene with a preference for C12:0-ACP from laurel was introduced into rapeseed. The transgenic lines showed an increase in dodecanoic acid content to 50% to 63%, respectively, with the normal growth of the rapeseed plants [78].

## 6. Transcription Factors in the Biosynthesis of Triacylglycerol

**Figure 3:**
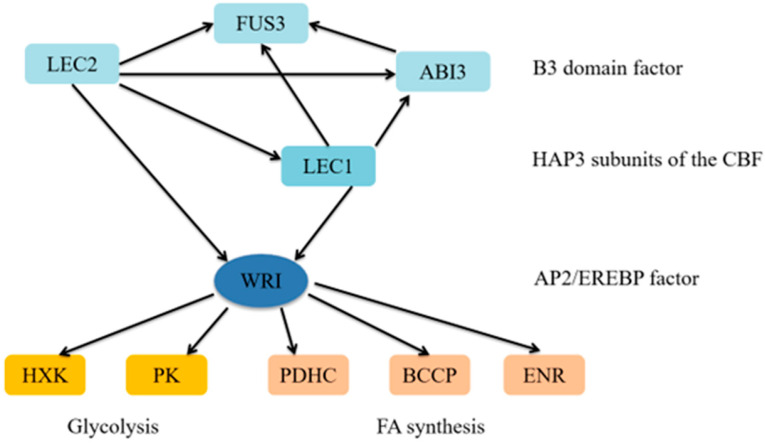
Transcriptional regulation model of triacylglycerol biosynthesis in plant seeds. Note: HXK: Hexokinase, PK: Pyruvate kinase, PDHC: Pyruvate dehydrogenase complex, BCCP: Biotin carboxyl carrier protein (second domain of ACCase), ENR: Enoyl-ACP reductase. Arrows indicate positive effects.

In the process of oil improvement, the regulation of a transcription factor involved in oil synthesis can influence a series of gene expressions within the metabolic pathway, leading to a significant increase in oil content. The regulation of certain transcription factors related to lipid synthesis and metabolism is depicted in Figure 3 [40].

### 6.1. Leaf Cotyledon (LEC)

Leaf cotyledon (LEC) is a key regulator in the process of embryogenesis and development. It controls many aspects of embryonic development and also plays an important role in the synthesis of fatty acids. *LEC2*, *FUS3*, and *ABI3* are members of the plant-specific B3 transcription factor superfamily, which together constitute the AFL (ABI3/FUS3/LEC2) family [79]. These transcription factors can regulate the development of mature seed characteristics, such as oil accumulation and cotyledon characteristics. *LEC2* and *LEC1* can upregulate each other’s expression [80]. They can also affect the expression of other transcription factors. It has been proven that *LEC1* and *LEC2* are upstream regulatory genes that affect *WRI* expression [81,82].

Li et al. cloned the cDNA of two members of *LEC1* in peanut [83]. It was found that the expression of *LEC1* was different at different stages of peanut seed development. Overexpression of the *AtLEC1* and *BnLEC1* genes greatly increased the fatty acid types and lipid content of transgenic *Arabidopsis* plants. Some studies have shown that LEC may increase fatty acid content by increasing the carbon flow to fatty acid synthesis [84]. Specifically, *LEC* may affect the oil content by regulating genes related to fatty acid synthesis, including genes encoding acetyl-CoA carboxylase, key enzymes controlling fatty acid synthesis, and genes involved in glycolysis and lipid accumulation [85].

### 6.2. WRINKLED1 (WRI)

WRINKLED1 (WRI) is a key transcription factor in the regulation of seed oil synthesis and accumulation. It can directly regulate glycolysis and fatty acid metabolism, thereby enhancing the overall expression level of fatty acid synthesis-related genes. The protein encoded by the gene contains two AP2/EREB domains, which control the transformation of sucrose into oil [86].

In 1998, a new *Arabidopsis* mutant named WRINKLED1 (WRI) was discovered for the first time. The oil content of the mutant decreased by 80%, the sucrose content increased, and the enzyme activities related to glycolysis were generally decreased [87]. Through cDNA microarray analysis, it was found that WRI mainly upregulated the key enzyme and fatty acid synthesis-related enzymes in the plastid glycolysis pathway at the transcriptional level to regulate oil accumulation. Similar to seed oil, WRI affects the oil content by regulating the expression of downstream genes such as pyruvate kinase (PK), pyruvate dehydrogenase (PDH), biotin carboxyl carrier protein L (CCPL), ENR, and hexokinase (HXK) in the mesocarp (non-seed) of oil plants.

In recent years, there have been many reports about the application of WRI in crop breeding. The *BnWRI* gene was overexpressed in Arabidopsis, and the seed oil content increased by 10–40% [88]. The *ZmWRI1* gene was overexpressed in maize, the oil content of transgenic progenies increased significantly, and the starch content did not decrease [89], and other agronomic traits were not significantly affected. The overexpression of *BnWRI1* in Brassica led to earlier flowering and increased seed oil content by 18–38% [90]. In addition, the ectopic expression of *WRI1* in the leaves of monocotyledon plant *Brachypodium distachyon* could increase the TAG content by 32.5 times and the free FA content by 2 times [91].

### 6.3. DNA Binding with One Finger (Dof)

DNA binding with one finger (Dof) is a kind of plant-specific transcription factor which is involved in the regulation of complex physiological activities in higher plants, such as the response of plants to hormones and light and the regulation of fatty acid synthesis [92]. There is a unique highly conserved domain of 52 amino acids in the N-terminal region of the Dof protein, and within this conserved domain, the CX2CX21CX2C motif forms a single zinc finger structure, in which one Zn^2+^ is covalently bound to four Cys residues [93].

In total, 28 Dof transcription factors were identified in soybean, and *GmDof4* and *GmDof11* were involved in oil synthesis. Studies have shown that *GmDof4* and *GmDofll* can bind to the promoter regions of ACCase and long chain acetyl-CoA synthetized genes, respectively, to activate the gene expression and increase the oil content of seeds [45].

## 7. The Strategies for Improving Oil Content through Precursor Transport and Multi-Gene Co-Regulation Involve Several Key Aspects

### 7.1. Transport of Triacylglycerol Precursors between Organelles

TAG synthesis sites involve multiple organelles, such as chloroplasts, endoplasmic reticulum, mitochondria, and matrix, among others. Acyl chains or lipids that are synthesized need to be transported from one site (organelle) to another. The transporters involved in the transport of acyl-CoA from plastids to the endoplasmic reticulum are not clear (existing studies are mainly based on yeast and mammalian models) [94,95]. The transport mechanisms of fatty acids are as follows: first, PC transports synthetic fatty acids from plastids to the endoplasmic reticulum. The mechanism may be that LPCAT catalyzes the synthesis of newly synthesized fatty acids into PC on the plastid membrane, and then the transport from plastids to the endoplasmic reticulum is realized by PC [96,97]. Second, ABC (ATP-binding cassette) transporters can also facilitate the transport process. The *Arabidopsis* ABCA family (a subfamily of the ABC family) has 12 members, and their function and mechanism are not fully understood [98]. Studies have shown that *ABCA9* mediates the transport of fatty acids from plastids to the endoplasmic reticulum, but the expression level of the *ABCA9* gene is lower than that of other members of the family, and the mechanism of action requires further study. Therefore, how to regulate the transport and storage of synthetic lipids and how to ensure their correct assembly are important issues in the regulation of lipid synthesis.

### 7.2. Multi-Gene Co-Regulation Based on Fatty Acid and Triglyceride Synthesis

Multi-gene co-regulation of the biosynthesis pathway is an important research direction in palm oil synthesis and metabolism. Plastid mRNA is translated by many polycistronic transcripts, and the transformed plastid genome may realize the expression of multiple genes within one transformation system [99]. Four genes of the DHA synthesis pathway were expressed in Arabidopsis plastids. Malonyl-CoA was successfully used to synthesize DHA in *Arabidopsis* seeds. Additionally, multiple vectors are an effective method for the simultaneous transformation of multiple genes. Multiple expression vectors are constructed from multiple key enzyme genes involved in the process of oil synthesis and metabolism, and the seed oil content is improved through transgenic methods. For example, five key enzyme genes of the EPA synthesis pathway are connected in the plant expression vector pCamBAR-5EC, and EPA synthesis and accumulation are detected in transgenic plants [100].

## 8. Discussion

Improving oil content in plants is a significant goal in crop breeding. The metabolism of fatty acids is closely related to plant stress resistance. Increasing the content of fatty acids, especially polyunsaturated fatty acids, plays an important role in maintaining cell membrane homeostasis. Currently, research on seed oil improvement mainly focuses on the fatty acid synthesis pathway and the triacylglycerol assembly pathway, with some enzymes and their catalytic reactions being relatively well understood [101,102,103]. However, the localization and mechanisms of action for some enzymes (such as the key enzymes KAR, HAD, and ENR involved in the carbon chain elongation cycle) in cells are not clear; plant oil synthesis involves multiple pathways such as sugar metabolism, pyruvate metabolism, and fatty acid metabolism, with numerous enzymes participating, and the interactions between these enzymes are not well understood [104]. The synthesis of fatty acids and the triacylglycerol (TAG) assembly processes are regulated at different levels (transcriptional regulation, post-transcriptional regulation, and metabolic regulation), and the regulatory networks are also unclear. Transcription factors can regulate the expression of multiple genes. Currently, transcription factors found to have significant effects on oil synthesis include *LEC1*, *LEC2*, *WRI1*, *Dof*, *ABI3*, *FUS3*, etc. [105]. Simultaneously, the construction of multiple vectors and their genetic transformation technology are effective technical solutions to ensure the coordinated expression of multiple gene metabolic pathways.

As an essential commodity in people’s lives, the study of the molecular mechanisms of metabolic regulation of plant oils and their applications is of great significance. In recent years, the release of plant genome data has provided new information for the study of plant genetic mechanisms. At the same time, the rapid development of sequencing technology has led to the maturation of transcriptome sequencing, microRNA sequencing, lncRNA sequencing, proteome sequencing, degradome sequencing, and methylation sequencing techniques, providing new platforms and means for studying the biochemical basis and regulatory mechanisms of plant oil formation [106,107,108]. Compared to animal oils, plant oils contain more polyunsaturated fatty acids, which are more beneficial to human health. With the continuous improvement of people’s living standards, the demand for plant oils is increasing, and the requirements for the quality of plant oils are also getting higher [109]. Regulating the metabolic process of plant seed oils through genetic engineering techniques is a reliable way to improve the content and quality of plant oils. Compared to traditional breeding, molecular-assisted breeding has the advantages of being directional and time-saving. Effectively combining molecular-assisted breeding with traditional breeding and applying it to production is a pressing issue for scientific breeders.

Plant oil biosynthesis is significantly influenced by various environmental factors such as temperature, humidity, and light, as well as by endogenous hormone signaling in plants. Temperature has a substantial impact on the accumulation of oils in plants, with high temperatures shortening the filling period of seeds, accelerating their dehydration and maturation, and reducing the content of stored substances in seeds. Temperature not only affects the amount of oil accumulation in seeds but also alters the composition of oils. In oil crops such as rapeseed and sunflower grown under high-temperature conditions, the levels of linoleic acid and α-linolenic acid decrease, while the content of oleic acid increases. Drought has a similar effect on plant oil accumulation to high temperatures; generally, drought leads to a decrease in oil content in seeds and an increase in the accumulation of saturated fatty acids. Light also influences the expression of key genes involved in oil synthesis, which in turn regulates the process of oil accumulation. Gibberellins (GAs) are an important class of plant hormones that play a role in regulating plant development processes such as germination, stem elongation, flowering, and dormancy. Additionally, key proteins in the GA signaling pathway, such as DELLA, are also involved in the regulation of seed oil content. In summary, the challenges and limitations of plant oil synthesis strategies in actual agricultural environments involve multiple aspects, including the adaptation of processing technologies, the research and application of new technologies, the advancement of breeding technologies, and the development strategies of organic agriculture.

In addition, energy shortage is a major issue facing the world, and plant oils are an important source of bioenergy. However, there are still many problems to be solved, such as the fact that plant TAGs cannot be directly used as raw materials for biofuels because the three hydroxyl groups on the glycerol in general plant TAGs are connected to fatty acids, known as long-chain TAGs, which have a high viscosity. In the seeds of *Euonymus alatus*, high concentrations of 3-acetyl-1,2-diacyl-snglycerols (acTAG) are mainly accumulated in the embryo and endosperm. These TAGs have an acetyl group connected to the third hydroxyl group instead of a long-chain fatty acid, which reduces their viscosity by 30% compared to long-chain TAGs, The gene *EaDAcT* encoding acTAG from Thunb was cloned and transformed into *Arabidopsis*. It was found that acTAG accounted for 40% of the total TAG in the seeds of the transgenic lines [109]

## 9. Conclusions

Lipid synthesis is a complex physiological and biochemical process. In recent years, there has been a deeper understanding of the biochemical pathways involved in lipid synthesis, and several key enzyme genes related to lipid synthesis have been successfully cloned and identified. This has led to some progress in using gene function technology to increase seed oil content or improve fatty acid composition. Currently, there is still limited knowledge about the transcriptional regulatory factors of lipid synthesis-related genes during seed development, and even less is known about the synergistic regulation between key enzymes in the synthesis and metabolic pathways. Additionally, there are many overlaps and intersections between lipid and membrane lipid synthesis. As an important site for cellular life activities, the synthesis and composition of lipids in the cell membrane are necessarily subject to precise regulation during its normal growth and development. We hypothesize that there may be undiscovered pathways for lipid synthesis, which could explain why some lipid synthesis-related gene mutants show little or no change in oil content. Exploring these unknown pathways will also provide more regulatory clues and pathways for increasing lipid content.

## Figures and Tables

**Table 1:** Functional validation of genes in the metabolism of fatty acid synthesis.

Genes Encoding the Key Enzymes and TFs	Function Confirmed in Original Species	Function Confirmed in Other Plants
Phosphoenolpyruvate carboxylase, PEPCase	CsPEPCase, HvPEPCase, RcPEPCase, BnPEPCi, OsPEPCi, AhPEPCi, GhPEPCase [23,24]	StPEPCase
Pyruvate dehydrogenase complex, PDHC	SoPDHC, PsPDHC [25], AtPDHC, BvPDHC [26]	
Acetyl CoA carboxylase, ACCase	AtACCase, GmACCase [27], RcACCase, AhACCase, GhACCase [28], NtACCase [29]	AtACCase [30]
β-ketoacyl-ACP synthase, KAS	AhKASI, BnKASIII [31], AtKASI, JcKASII [32]	
β-ketoacyl-ACP reductase, KAR	EgKAR, PdKAR [33], AhKR [34], GhKAR [35]	
Hydroxyacyl-ACP dehydrase, HAD	EgHAD, PdHAD [33], AhHD [34], GhHAD [35]	
Enoyl-ACP reductase, ENR	EgENR, PdENR [33], AhER, GhENR [35]	
Acyl-ACP thioesterase, FAT	AhFATB, AtFAT, RcFAT	LnFAT [29]
Glyceral 3-phosphate acyltransferase, GPAT	AtGPAT5, AtGPAT4, AtGPAT6	MaGPAT
Diacylglycerol acyltransferase, DGAT	EgDGAT2 [33], AtDGAT1 [36], OeDGAT2 [37]	UrDGAT [38], NcDGAT2 [39]
Leafy cotyledon, LEC	AtLEC1 [40], AhLEC1, AtLEC2 [41], ZmLEC1 [42]	
Transcription factor WRINKLED1, WRI	EgWRI1 [33], ZmWRI1 [42], AtWRI1 [43]	BnWRI1 [43]
DNA binding with one finger, Dof	ZmDof [44]	GmDof [45]

Cs: *Citrus sinensis*, Hv: *Hordeum vulgare*, Rc: *Ricinus communis*, Bn: *Brassica napus*, Os: *Oryza sativa*, Ah: *Arachis hypogaea*, Gh: *Gossypium hirsutum*, St: *Solanum tuberosum*, So: *Spinacia oleracea*, Ps: *Pisum sativum*, At: *Arabidopsis thaliana*, Bv: β vulgaris, Gm: *Glycine max*, Nt: *Nicotiana tabacum*, Jc: *Jatropha curcas*, Eg: *Elaeis guineensis*, Pd: *Phoenix dactylifera*, Ln: *Laurus nobilis*, Ma: *Malus asiatica*, Oe: *Olea europaea*, Ur: *Umbelopsis ramanniana*, Nc: *Neurospora crassa*, Zm: *Zea mays* L.
